# Pressure-Dependent Stability of Imidazolium-Based Ionic Liquid/DNA Materials Investigated by High-Pressure Infrared Spectroscopy

**DOI:** 10.3390/ma12244202

**Published:** 2019-12-13

**Authors:** Teng-Hui Wang, Min-Hsiu Shen, Hai-Chou Chang

**Affiliations:** Department of Chemistry, National Dong Hwa University, Hualien 974, Taiwan; 810712101@gms.ndhu.edu.tw (T.-H.W.); 410412053@gms.ndhu.edu.tw (M.-H.S.)

**Keywords:** ionic liquid, high pressure, IR

## Abstract

1-Butyl-3-methylimidazolium hexafluorophosphate ([C_4_MIM][PF_6_])/DNA and 1-methyl-3-propylimidazolium hexafluorophosphate ([C_3_MIM][PF_6_])/DNA mixtures were prepared and characterized by high-pressure infrared spectroscopy. Under ambient pressure, the imidazolium C^2^–H and C^4,5^–H absorption bands of [C_4_MIM][PF_6_]/DNA mixture were red-shifted in comparison with those of pure [C_4_MIM][PF_6_]. This indicates that the C^2^–H and C^4,5^–H groups may have certain interactions with DNA that assist in the formation of the ionic liquid/DNA association. With the increase of pressure from ambient to 2.5 GPa, the C^2^–H and C^4,5^–H absorption bands of pure [C_4_MIM][PF_6_] displayed significant blue shifts. On the other hand, the imidazolium C–H absorption bands of [C_4_MIM][PF_6_]/DNA showed smaller frequency shift upon compression. This indicates that the associated [C_4_MIM][PF_6_]/DNA conformation may be stable under pressures up to 2.5 GPa. Under ambient pressure, the imidazolium C^2^–H and C^4,5^–H absorption bands of [C_3_MIM][PF_6_]/DNA mixture displayed negligible shifts in frequency compared with those of pure [C_3_MIM][PF_6_]. The pressure-dependent spectra of [C_3_MIM][PF_6_]/DNA mixture revealed spectral features similar to those of pure [C_3_MIM][PF_6_]. Our results indicate that the associated structures of [C_4_MIM][PF_6_]/DNA are more stable than those of [C_3_MIM][PF_6_]/DNA under high pressures.

## 1. Introduction

Deoxyribonucleotide acid (DNA) molecules, which are double-helical biopolymers comprised of attached nucleotides, are well known to serve as genetic information carriers [1,2,3,4,5]. Because of the Watson–Crick interaction, that is, hydrogen bonding between nucleobase pyrimidine and purine, and base–base interaction (π–π stacking), DNA molecules can exist in stable conformations at ambient temperatures in cell nuclei [2,3]. The expected applications of DNA are far more than what current research can offer, because evidence increasingly suggests that DNA molecules are programmable in well-defined structures for 3D designing and topology [4,5]. Shih et al. [6] have discovered that ss-DNA can be configured into double-helical struts linked at the branched junctions, and two kinds of noncovalent motifs (double-crossover and paranemic-crossover struts) facilitate the formation of triangulated objects, such as tetrahedra or octahedra. Several studies [5,6,7,8,9] have also clarified that the specific associations and hydrophobic interactions make nucleic acid a promising candidate for the production of designable building blocks via self-assembly. In addition, various investigations show that the behaviors of self-assembly and electrostatic trapping of DNA can be used in drug delivery [8] and nanoelectronics [9].

Ionic liquids (ILs) are recognized as superb green solvents because of their low vapor pressure and recyclability, as well as several other unique characteristics [10,11,12,13,14,15], such as high electric conductivity and being great stabilizers for biomaterials and nucleic acids [16]. Particularly, ILs containing imidazolium-ring cation are considered excellent biocompatible (or bioactive) substances, amorphous liquids with designable subunits, and ion-conductive materials [10,11,12,13]. The alkyl side chain of an imidazolium cation may participate in aggregation and association with anions in bulk ILs, although the hydrophilic and Coulomb forces remain the dominant interactions [11,12,13,14,15]. On top of that, several reports [17,18,19,20,21,22,23,24] show that the hydrophobic alkyl chains can stabilize biopolymers like nucleic acids via forming the association through electrostatic interactions between the DNA molecule and cations of IL.

DNA molecules can associate with cations of IL via the negatively charged backbone and hydrophobic groove interaction of DNA [19,20,21,22,23,24]. The cluster structure of ILs can be disturbed by mixing with an additive-like DNA via electrostatic interaction between the cation head group of ILs and phosphate anion of DNA and hydrophobic association between the alkyl part of IL cation and the groove region or nitrogenous base of DNA [19,20,21,22,23,24]. Part of base pairs (pyrimidine and purine), which interact via hydrogen bonding and stacking, are easily attracted to alkyl tail of ILs with noncovalent interaction, hydrophobic interaction, or van der Waals force. Several researchers [19,20,21,22] have suggested that the chemical structure of the cationic head group of ILs determines the electrostatic interaction to a certain extent, whereas the alkyl chain length of the cation is responsible for the stability of groove association. Ghoshdastidar et al. [25] have reported that ILs can rescue DNA by forming a complex via specific trapping force in water solutions. Moreover, as the lifetime of DNA in aqueous solutions is strictly limited by hydrolysis, Tateishi-Karimata et.al. [16] suggested that the more stable the DNA–IL association is, the longer storage time of DNA can become. It is currently deemed that the stability of DNA can be attributed to five factors: the Watson–Crick interaction, base pair stacking, conformational entropy, hydration, and cation binding [16]. The conformation of DNA–IL complexes in an aqueous solution or in neat ILs reveals that the durability depends on the hydration and cation binding, which can be adjusted by varying ILs and constituent concentrations. The ILs that can spontaneously self-assemble and specifically attach to groove and backbone structures allow to skillfully control the DNA structure [19,20,25].

High pressure allows to force ions or molecules into close-range to enhance the interactions between them, which makes it a useful and effective technique. Previous studies [26,27,28] revealed that pure ILs have an anisotropic cluster structure, and the hydrogen-bonding network can be easily disturbed by adding various materials under high-pressure conditions. Deemyad et al. [29,30,31] concluded that pressure can induce superconductivity and electronic properties modification of Cu_2_I_2_Se_6_ by a structural change. The electronic responses, optoelectronic properties, energy band gaps, and crystal structures of different materials, such as semiconductors [29], perovskites [30], and piezochromic materials [31], can be varied by compression. For example, IL-β-cyclodextrin (IL-β-CD) associated forms under low pressures can be turned to dissociated structures with the pressure increase [26]. The studies of pressure-dependent confinement effect of ILs indicated that interfacial IL–silica interactions tend to weaken the cation–anion interactions under high pressure [27]. In this study, we demonstrate that high pressure may provide a unique insight into the stabilization of DNA–IL associations and key interactions between DNA and IL.

## 2. Materials and Methods

Samples were prepared using deoxyribonucleic acid sodium salt from salmon testes (DNA, Sigma-Aldrich, St. Louis, MO, USA), 1-butyl-3-methylimidazolium hexafluorophosphate ([C_4_MIM][PF_6_], ≥97.0%, Sigma-Aldrich), and 1-methyl-3-propylimidazolium hexafluorophosphate ([C_3_MIM][PF_6_], 99.0%, UniRegion Bio-Tech, Taoyuan, Taiwan). IL ([C_3_MIM][PF_6_] or [C_4_MIM][PF_6_]) and DNA aqueous solution (1.6 wt%) were mixed with the volume ratio of 1:1, and the mixture was sonicated at 30 °C for 30 min. Then, the mixture was centrifuged at 12,000 rpm for 10 min. The sample solution was separated into two phases. We note that [C_3_MIM][PF_6_] and [C_4_MIM][PF_6_] are hydrophobic (water-insoluble) ionic liquids. The lower IL phase was removed, and the upper aqueous solution was collected and dried to obtain the DNA–IL mixtures.

High pressure (up to ~2 GPa) was generated using a diamond anvil cell (DAC) with a diamond culet size of 0.6 mm. The DAC contained two type-IIa diamonds, which are suitable for mid-infrared (mid-IR) measurements. IR spectra were measured using a Fourier-transform (FT) spectrophotometer (Spectrum RXI, Perkin-Elmer, Naperville, IL, USA) equipped with a lithium tantalite detector. To enhance the intensity of passed infrared beam, a five-beam condenser was combined with the FT spectrometer. To eliminate the influence of absorption of the diamond anvils, the absorption spectra of the DAC were measured first and subtracted from those of the samples. A 0.25 mm thick Inconel gasket with a 0.3 mm diameter hole was prepared to hold the sample. To reduce the absorbance of the samples, transparent CaF_2_ crystals were placed into the holes and compressed prior to inserting the samples. A resolution of 4 cm^−1^ (data point resolution of 2 cm^−1^) and 1000 scans were chosen for the high pressure data. Pressure calibration was performed following Wong’s method [32,33]. The spectra of samples at ambient pressure were obtained by putting samples in a cell with two CaF_2_ windows. To obtain the amount of water in the DNA mixtures (ca. 9 wt%), a moisture analyzer (MS-70, A&D Company, Tokyo, Japan) was used.

## 3. Results and Discussion

**Figure 1:**
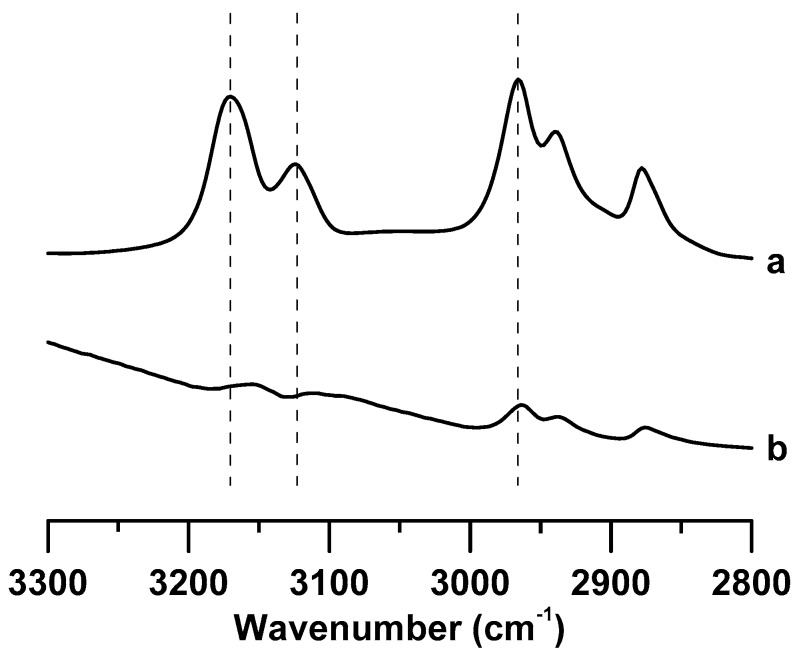
IR spectra of (**a**) pure [C_4_MIM][PF_6_] and (**b**) the [C_4_MIM][PF_6_]/DNA mixture recorded under ambient pressure.

Figure 1 shows the IR spectra of (a) pure [C_4_MIM][PF_6_] and (b) [C_4_MIM][PF_6_]/DNA mixture recorded under ambient pressure. The IR spectrum of pure [C_4_MIM][PF_6_] in Figure 1a shows two imidazolium peaks at 3124 and 3169 cm^−1^, which correspond to the vibrational absorption bands of C^2^–H and C^4,5^–H on the imidazolium ring, respectively [26,27,28]. The other three bands in the region of 2850–3000 cm^−1^ in Figure 1a are assigned to the alkyl C–H vibrational modes on the cation tail of pure [C_4_MIM][PF_6_] [26,27,28]. The curve fitting and deconvolution of pure ionic liquids spectra were performed with Lorentzian peaks. In Figure 1b, the baseline absorption bands in the region of 3000–3300 cm^−1^ may be attributed to the hydrogen-bonded O–H or N–H absorption bands. Comparing pure [C_4_MIM][PF_6_] (Figure 1a) with the [C_4_MIM][PF_6_]/DNA mixture (Figure 1b), evident differences in the spectral features of the C^2^–H and C^4,5^–H bands can be observed. The C^2^–H and C^4,5^–H absorption bands of the [C_4_MIM][PF_6_]/DNA mixture are slightly red-shifted to 3113 and 3154 cm^−1^, respectively, compared with those of pure [C_4_MIM][PF_6_] at ambient pressure (Figure 1). On the other hand, the alkyl C–H absorption bands in Figure 1b do not reveal a significant frequency shift for the [C_4_MIM][PF_6_]/DNA mixture at ambient pressure. The results in Figure 1 indicate that DNA molecules may interact with the imidazolium ring of [C_4_MIM][PF_6_], and the local structures of C^2^–H and C^4,5^–H are influenced by DNA. We noticed that the imidazolium C–H absorption bands of [C_4_MIM][PF_6_]/DNA are relatively broad in bandwidth in Figure 1b compared with those of pure [C_4_MIM][PF_6_] in Figure 1a. The imidazolium C–H local structures of [C_4_MIM][PF_6_]/DNA may exist in multiple imidazolium C–H–DNA conformations such as strong complexation, weak association, and mild (or negligible) interactions. The curve fitting and deconvolution of imidazolium C–H bands for [C_4_MIM][PF_6_]/DNA were performed with Gaussian functions, and the spectra were subtracted by a straight line prior to deconvolution to avoid the interference from the baseline. Figure S1 (see Supplementary Materials) shows the infrared spectra of (a) pure [C_4_MIM][PF_6_] and (b) [C_4_MIM][PF_6_] with saturated water. As revealed in Figure S1, the C–H absorptions are almost identical for pure [C_4_MIM][PF_6_] and [C_4_MIM][PF_6_] with saturated water owing to the hydrophobicity of [C_4_MIM][PF_6_]. Thus, the peak shifts observed in Figure 1 may be attributed to the interactions between IL and DNA instead of the interactions with residual water molecules.

**Figure 2:**
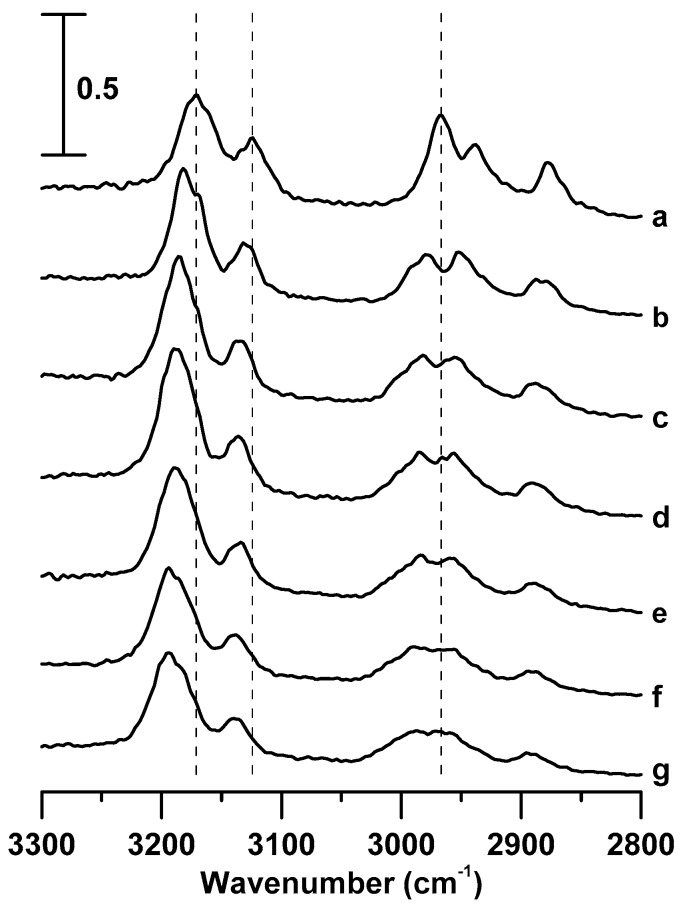
IR spectra of pure [C_4_MIM][PF_6_] obtained at (**a**) ambient pressure and (**b**) 0.4, (**c**) 0.7, (**d**) 1.1, (**e**) 1.5, (**f**) 1.8, and (**g**) 2.5 GPa.

Figure 2 shows the IR spectra of pure [C_4_MIM][PF_6_] obtained at (a) ambient pressure and (b) 0.4, (c) 0.7, (d) 1.1, (e) 1.5, (f) 1.8, and (g) 2.5 GPa. When the pressure was increased to 0.4 GPa (Figure 2b), the C^4,5^–H, C^2^–H, and alkyl C–H bands underwent an extraordinary blue shift to 3182, 3132, and 2978 cm^−1^, respectively. In addition, the alkyl C–H absorption bands at approximately 2978 and 2950 cm^−1^ in Figure 2b show the decrease in absorption ratio (I_2978_/I_2950_) with the increase of pressure from ambient (Figure 2a) to 0.4 GPa (Figure 2b). These results indicate that the pressure-enhanced interactions may disturb the hydrogen-bond network and change the local structures. With the further increase of pressure from 0.4 to 2.5 GPa (Figure 2b–g), the C–H absorption bands (C^4,5^–H, C^2^–H, and alkyl C–H) show mild blue shifts and broadening of the bandwidths. The effect can be understood as follows: the applied pressure forces [C_4_MIM]^+^ cations and [PF_6_]^−^ anions to move closer, which enhances the interaction between them.

**Figure 3:**
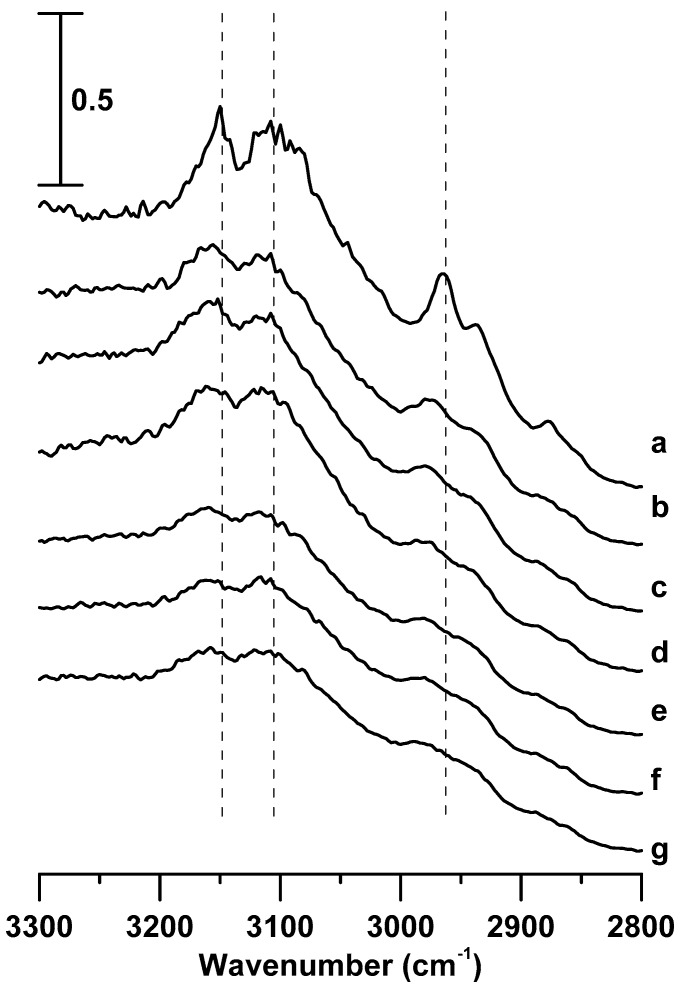
IR spectra of the [C_4_MIM][PF_6_]/DNA mixture obtained at (**a**) ambient pressure and (**b**) 0.4, (**c**) 0.7, (**d**) 1.1, (**e**) 1.5, (**f**) 1.8, and (**g**) 2.5 GPa.

Figure 3 shows the IR spectra of the [C_4_MIM][PF_6_]/DNA mixture obtained at (a) ambient pressure and (b) 0.4, (c) 0.7, (d) 1.1, (e) 1.5, (f) 1.8, and (g) 2.5 GPa. As shown in Figure 3, baseline absorption is present for the [C_4_MIM][PF_6_]/DNA mixture in all of the spectra (Figure 3a–g), which is attributed to the absorption bands of hydrogen-bonded O–H or N–H. With the increase of pressure to 0.4 GPa, the C^2^–H and C^4,5^–H absorption bands reveal mild blue shifts to 3115 and 3158 cm^−1^, respectively (Figure 3b). The alkyl C–H bands in the range from 2850 to 3000 cm^−1^ are also blue-shifted because of the pressure increase (Figure 3b). In agreement with our experimental results, several researchers [16,19,20,24,25] suggested that [C_4_MIM]^+^ cations can be easily bound into the minor groove structure of DNA molecules by the electrostatic force and van der Waals interactions. In comparison with pure [C_4_MIM][PF_6_] at the pressure of 0.4 GPa (Figure 2b), the C^2^–H and C^4,5^–H absorption bands of [C_4_MIM][PF_6_]/DNA mixture at 0.4 GPa (Figure 3b) show smaller frequency shifts under compression. Previous studies [26,27,28] revealed that high pressure can enhance the cluster structure interaction and lead to a band frequency shift. The hydrogen-bond network of an IL cluster structure may be disrupted, as some added molecules (such as DNA) may disturb the associations of ILs or cut large aggregations to small pieces. With the further increase of pressure from 0.4 to 2.5 GPa (Figure 3b–g), the C–H bands do not show significant frequency shifts; however, the C–H absorption bands reveal subtle-continuous band-broadening. DNA may protect imidazolium cations from approaching molecules or ions under applied pressure, which makes the associated [C_4_MIM][PF_6_]/DNA structure stable under up to 2.5 GPa pressure.

**Figure 4:**
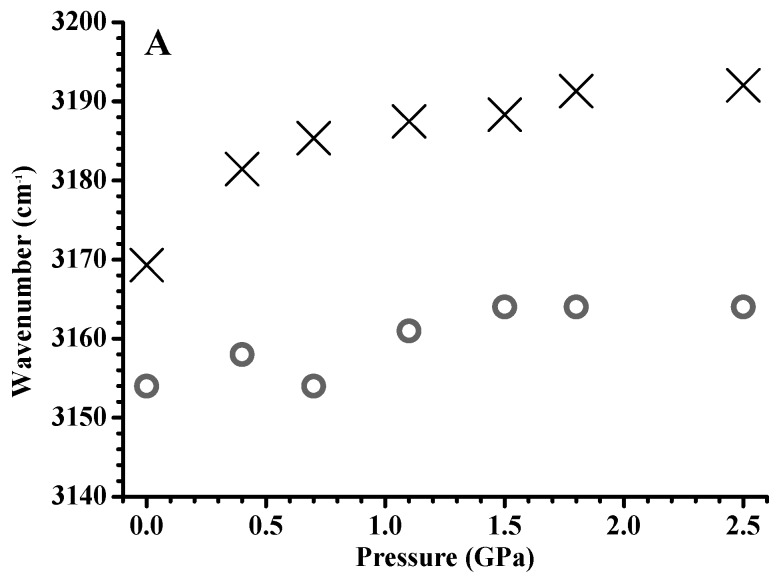

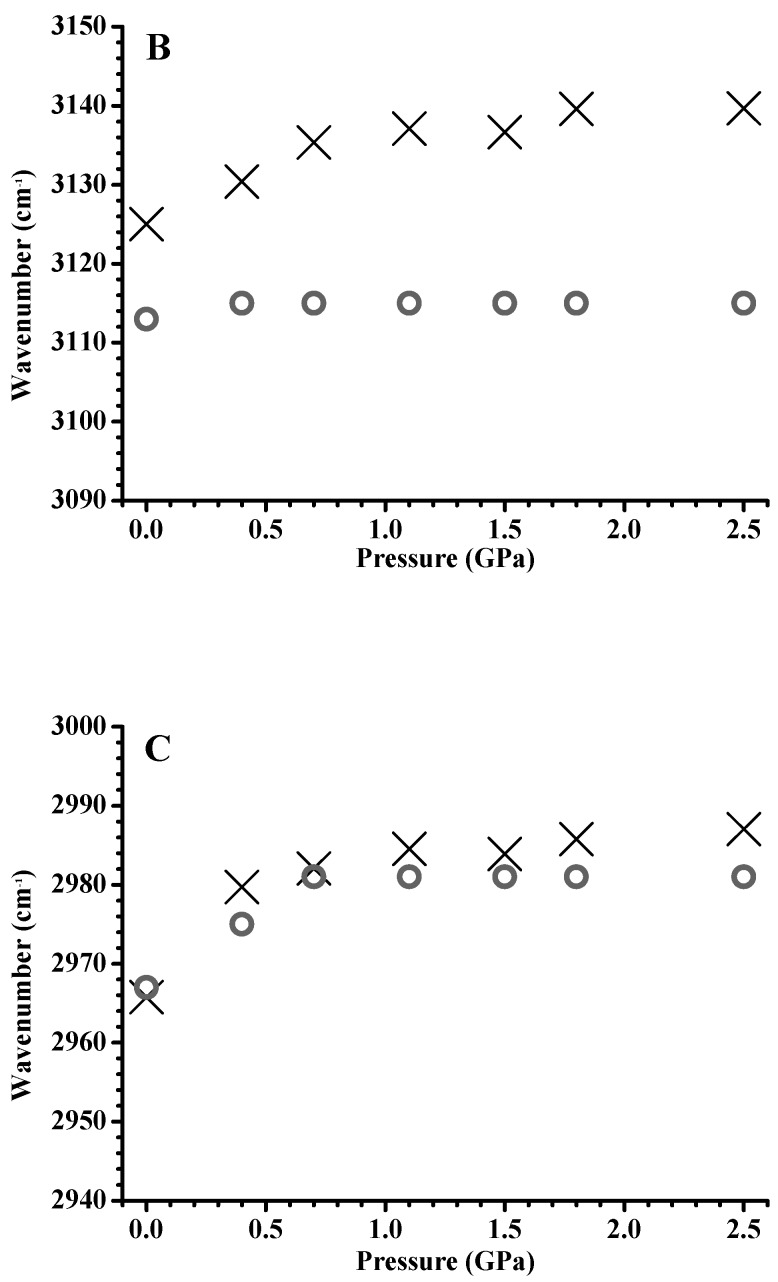
Pressure dependence of C^4,5^–H (**A**), C^2^–H (**B**), and alkyl C–H (**C**) stretching frequencies of pure [C_4_MIM][PF_6_] (cross) and the [C_4_MIM][PF_6_]/DNA mixture (circles).

Figure 4 shows the pressure dependence of C–H stretching frequencies of pure [C_4_MIM][PF_6_] and the [C_4_MIM][PF_6_]/DNA mixture. The imidazolium C^4,5^–H and C^2^–H stretching bands for pure [C_4_MIM][PF_6_] (Figure 4A,B) show drastic blue shifts in the pressure range from ambient to 0.7 GPa and mild blue shifts in the pressure range from 0.7 to 2.5 GPa. Previous studies indicated [26,27,28] that high pressure can easily shorten the imidazolium C–H bonds by the enhancement of weak C–H hydrogen bonding, which leads to a blue shift. Blue shifts of the pure [C_4_MIM][PF_6_] C–H stretching band in Figure 4A,B may be related to the C–H…F interaction between imidazolium C–H bands (C^4,5^–H and C^2^–H) and [PF_6_]^−^ under increased pressure. For the C^4,5^–H and C^2^–H imidazolium vibrational bands of the [C_4_MIM][PF_6_]/DNA mixture (Figure 4A,B), mild band shifts were observed as the pressure was increased from ambient to 2.5 GPa. The imidazolium C–H band shifts for the [C_4_MIM][PF_6_]/DNA mixture do not display similar trends to the results of pure [C_4_MIM][PF_6_] (Figure 4A,B). The DNA molecules may somehow prevent ILs from forming the hydrogen-bond network at high pressures. In other words, pure [C_4_MIM][PF_6_] may form large cluster structures under high pressures, while the stable [C_4_MIM][PF_6_]/DNA structures prevent [C_4_MIM][PF_6_] from aggregation. The alkyl C–H bands of the cation for pure [C_4_MIM][PF_6_] in Figure 4C show blue shifts at the pressure increase from ambient to 0.7 GPa and slight frequency shifts in the pressure range from 0.7 to 2.5 GPa. While the alkyl C–H absorption bands for the [C_4_MIM][PF_6_]/DNA mixture (Figure 4C) underwent blue shifts under pressures below 0.7 GPa, the alkyl C–H bands showed no significant frequency shifts at pressures above 0.7 GPa. The vibrational-band shifts of the C^4,5^–H and C^2^–H absorption bands of mixtures in Figure 4A,B show different trends in comparison with those for the alkyl C–H bands in Figure 4C. This indicates that local associations between imidazolium C–H (C^4,5^–H and C^2^–H) and DNA are dominant in the mixture, and the interactions between alkyl C–H and DNA are not sufficiently strong to fully disturb the alkyl C–H–anion interactions under high pressures. The band-shift differences under high pressures may be attributed to the differences in the interaction magnitudes of electrostatic association, hydrophobic interaction, and van der Waals force [1,16]. The IR spectra of [C_4_MIM][PF_6_]/DNA (ambient and cycled back to ambient) are shown in Figure S2 (see Supplementary Materials), and the spectra are reversible upon pressure cycling. Pressure-induced reversible unfolding of biomolecules has drawn the attention of researchers [34]. Pressure denaturation leads to a more controlled perturbation to the structures of biomolecules than chemical or temperature denaturation. High-pressure NMR (with high resolution) may provide the sensitive approach in studies of the pressure-induced denaturation problems [34].

**Figure 5:**
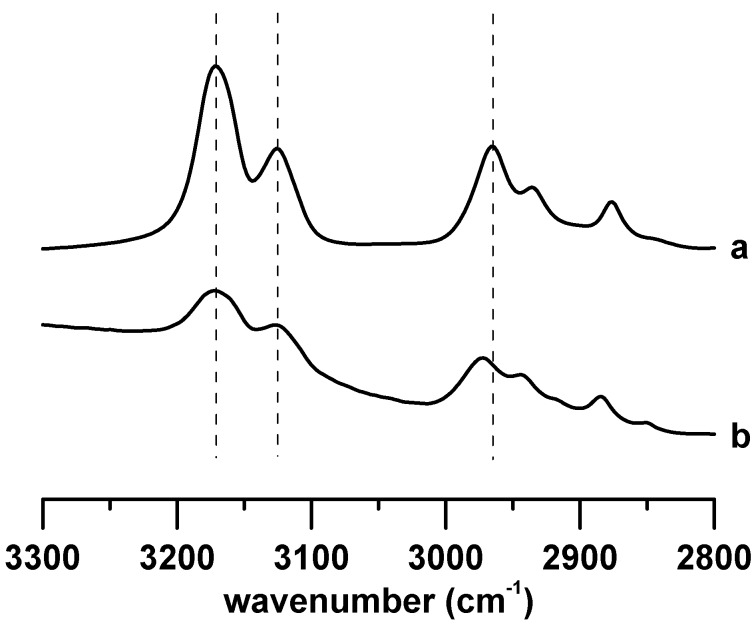
IR spectra of (**a**) pure [C_3_MIM][PF_6_] and (**b**) the [C_3_MIM][PF_6_]/DNA mixture recorded under ambient pressure.

To investigate the interactions between DNA and ILs with various alkyl-chain lengths, combining [C_3_MIM][PF_6_] and DNA may provide more hints on the effect of the DNA–IL association. Figure 5 shows the IR spectra of (a) pure [C_3_MIM][PF_6_] and the (b) [C_3_MIM][PF_6_]/DNA mixture recorded under ambient pressure. The IR spectrum of pure [C_3_MIM][PF_6_] in Figure 5a reveals the C–H absorption bands at 3172 (C^4,5^–H), 3124 (C^2^–H), and 2965 (alkyl C–H) cm^−1^. The C–H absorption frequencies of pure [C_3_MIM][PF_6_] in Figure 5a are similar to those of pure [C_4_MIM][PF_6_] in Figure 1a at ambient pressure. In Figure 5b, the imidazolium C–H (C^4,5^–H and C^2^–H) band frequencies of the [C_3_MIM][PF_6_]/DNA mixture show slight frequency shifts compared with those of pure [C_3_MIM][PF_6_] in Figure 5a. The imidazolium C–H frequency shifts induced by DNA in Figure 5 are fairly small in comparison with those for [C_4_MIM][PF_6_]/DNA in Figure 1. Thus, the weak associated configurations with DNA may be the dominant species for the [C_3_MIM][PF_6_]/DNA mixture instead of the species of strong complexation for [C_4_MIM][PF_6_]/DNA. It should be noted that the alkyl C–H band at 2972 cm^−1^ of the [C_3_MIM][PF_6_]/DNA mixture in Figure 5b shows a mild blue shift in comparison with the alkyl C–H absorption band of pure [C_3_MIM][PF_6_] in Figure 5a. The band shifts of alkyl C–H for pure [C_3_MIM][PF_6_] and the [C_3_MIM][PF_6_]/DNA mixture in Figure 5 may be attributed to the local structure changes of the alkyl C–H groups induced by the presence of DNA molecules.

**Figure 6:**
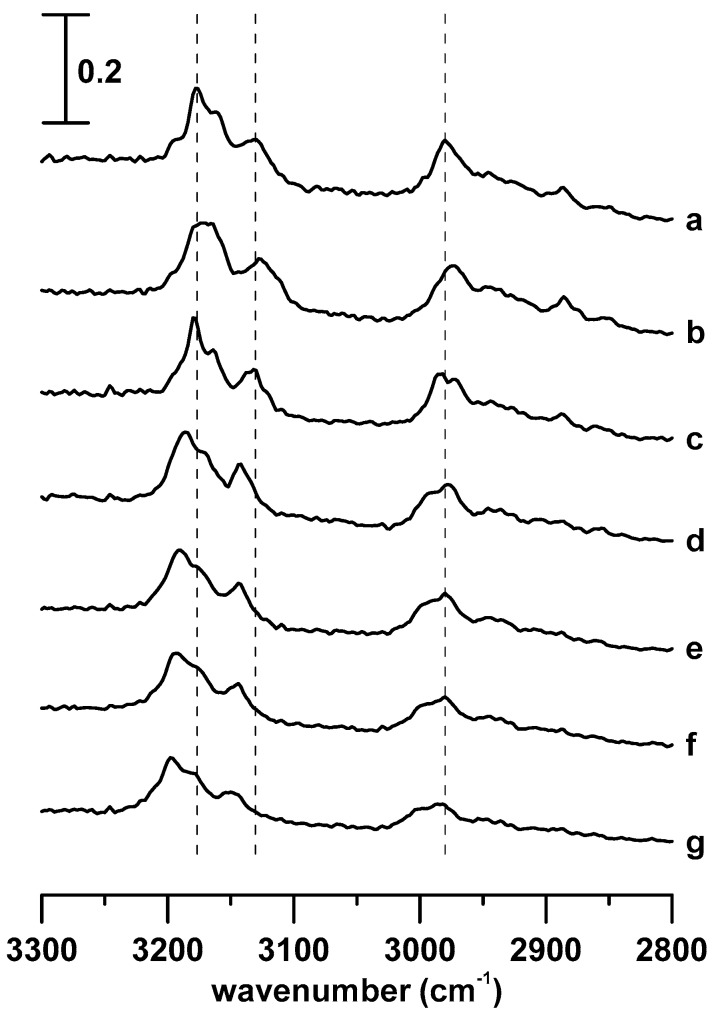
IR spectra of pure [C_3_MIM][PF_6_] obtained at (**a**) ambient pressure and (**b**) 0.4, (**c**) 0.7, (**d**) 1.1, (**e**) 1.5, (**f**) 1.8, and (**g**) 2.5 GPa.

**Figure 7:**
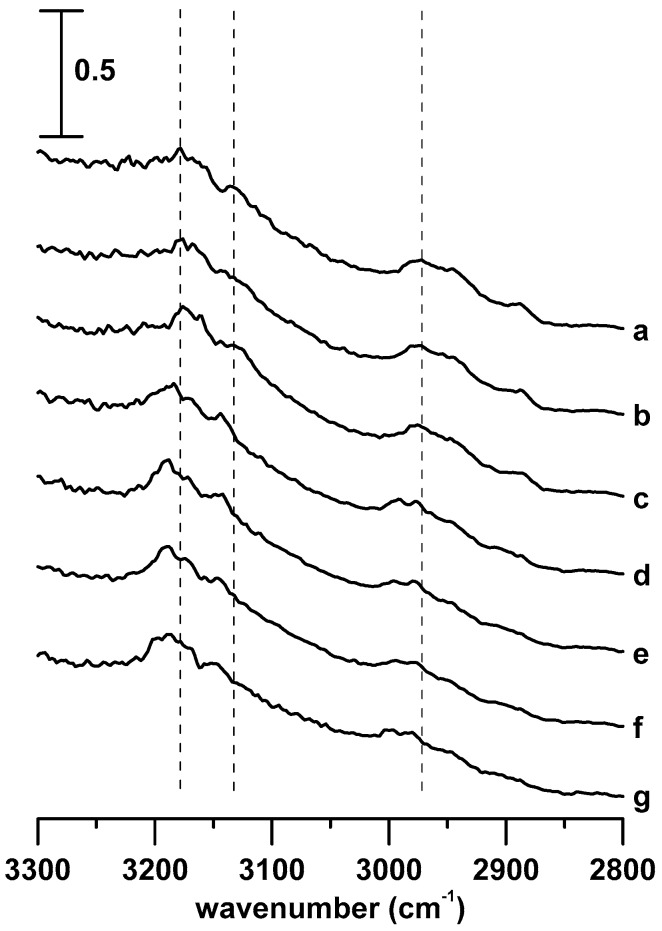
IR spectra of the [C_3_MIM][PF_6_]/DNA mixture obtained at (**a**) ambient pressure and (**b**) 0.4, (**c**) 0.7, (**d**) 1.1, (**e**) 1.5, (**f**) 1.8, and (**g**) 2.5 GPa.

The pressure-dependent IR spectra of pure [C_3_MIM][PF_6_] and the [C_3_MIM][PF_6_]/DNA mixture are shown in Figure 6 and Figure 7, respectively. The C–H absorption bands of pure [C_3_MIM][PF_6_] and the [C_3_MIM][PF_6_]/DNA mixture display similar band shifts and spectral features upon compression.

**Figure 8:**
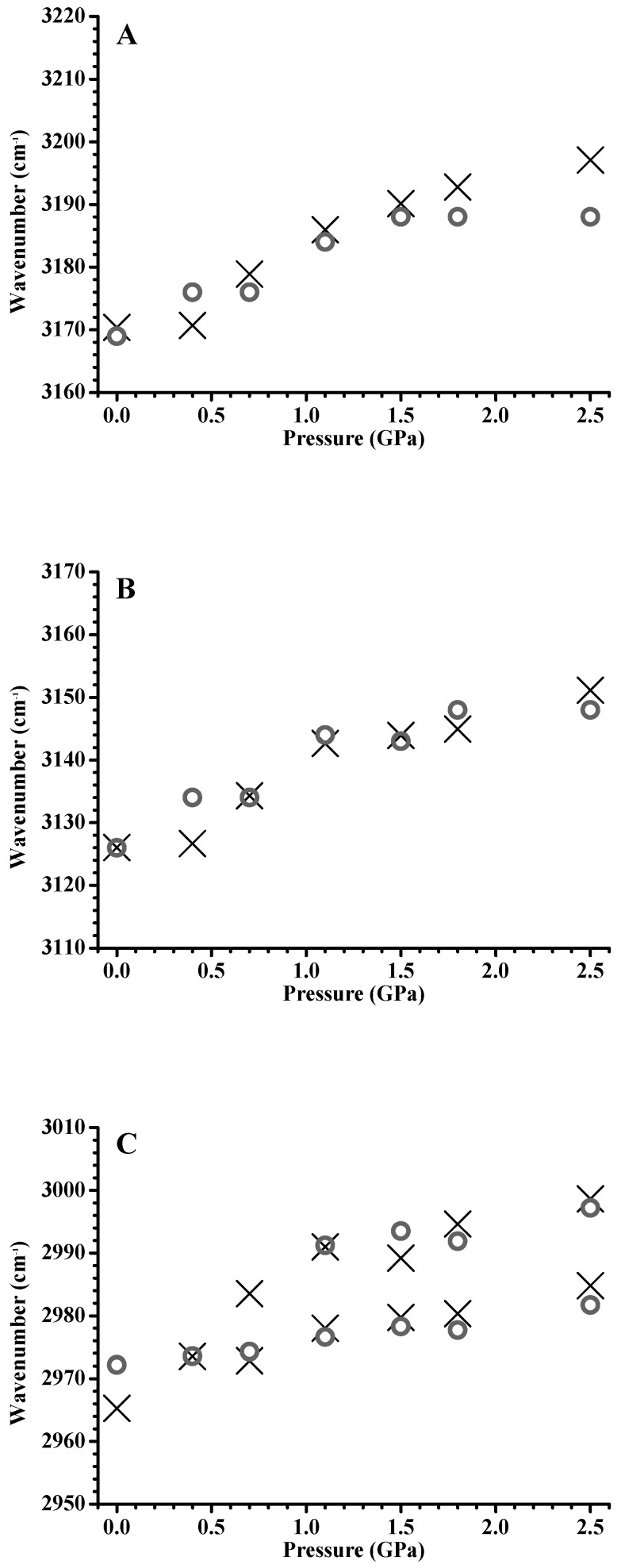
Pressure dependence of the C^4,5^–H (**A**), C^2^–H (**B**), and alkyl C–H (**C**) stretching frequencies of pure [C_3_MIM][PF_6_] (cross) and the [C_3_MIM][PF_6_]/DNA mixture (circles).

Figure 8 shows the pressure dependence of the C–H stretching frequencies of pure [C_3_MIM][PF_6_] and the [C_3_MIM][PF_6_]/DNA mixture. The C^4,5^–H and C^2^–H stretching bands (Figure 8A,B) of pure [C_3_MIM][PF_6_] and the [C_3_MIM][PF_6_]/DNA mixture show similar band-shift tendencies under compression. The imidazolium band-shift results of [C_3_MIM][PF_6_]/DNA in Figure 8A,B are remarkably different from those of [C_4_MIM][PF_6_]/DNA in Figure 4A,B. The differences may be attributed to the stronger association between [C_3_MIM]^+^ and [PF_6_]^−^ caused by more symmetric and easier packing of [C_3_MIM]^+^ than that of [C_4_MIM]^+^. Namely, cations with short alkyl side chain may favor the local cation–anion structures at high pressures. Cations with a longer alkyl side chain may lead to larger binding forces with DNA. This observation is consistent with the arguments reported in the literature [22,35]. In other words, the difference in alkyl side chain lengths may cause various effects on the stabilization of IL/DNA associations at high pressures. Figure 8C shows the pressure dependence of alkyl C–H band shifts for pure [C_3_MIM][PF_6_] and the [C_3_MIM][PF_6_]/DNA mixture. The splitting of the alkyl C–H band for pure [C_3_MIM][PF_6_] occurs at the pressure of 0.7 GPa. The splitting may be attributed to the phase transition and pressure-induced local structural organization. For [C_3_MIM][PF_6_]/DNA, the pressure required to split the alkyl C–H band changed to 1.1 GPa (Figure 6C). Thus, the presence of DNA indeed disturbs the local structure of alkyl C–H groups of cations in a [C_3_MIM][PF_6_]/DNA mixture.

## 4. Conclusions

In this study, high-pressure measurements were performed to investigate the stabilization of DNA–IL associations. Pressure-dependent studies revealed that [C_4_MIM][PF_6_]/DNA association is stable up to the pressure of 2.5 GPa. DNA molecules prevent [C_4_MIM][PF_6_] from forming the hydrogen-bond network under high pressures. Nevertheless, the pressure-dependent IR spectra of pure [C_3_MIM][PF_6_] and the [C_3_MIM][PF_6_]/DNA mixture display similar band shifts and spectral features. The alkyl C–H side chain may play a non-negligible role in IL/DNA associations. Cations with longer alkyl side chain can possibly provide stronger binding interactions with DNA.

## Supplementary Materials

The following are available online at https://www.mdpi.com/1996-1944/12/24/4202/s1, Figure S1: Infrared spectra of (a) pure [C_4_MIM][PF_6_] and (b) [C_4_MIM][PF_6_] with saturated water. Figure S2: IR spectra of [C_4_MIM][PF_6_]/DNA obtained at (a) ambient pressure and (b) cycled back to ambient.
