# Supplementary material for: Pressure-Dependent Stability of Imidazolium-Based Ionic Liquid/DNA Materials Investigated by High-Pressure Infrared Spectroscopy
Source: Materials (Basel). 2019 Dec 13;12(24):4202. doi: 10.3390/ma12244202 (PMC6947093; doi:10.3390/ma12244202)
Supplement: Supplementary file 1 [file materials-12-04202-s001.pdf]

materials

Supplementary Materials

Pressure-Dependent Stability of Imidazolium-Based Ionic Liquid/DNA Materials Investigated by High-Pressure Infrared Spectroscopy

Teng-Hui Wang, Min-Hsiu Shen, Hai-Chou Chang

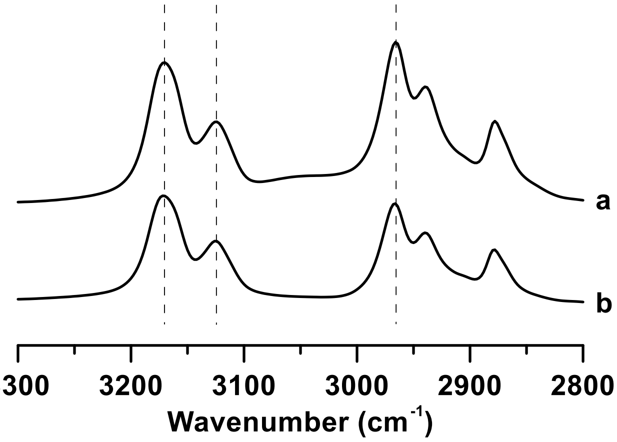

Figure S1. Infrared spectra of (a) pure [C_4MIM][PF_6] and (b) [C_4MIM][PF_6] with saturated water.

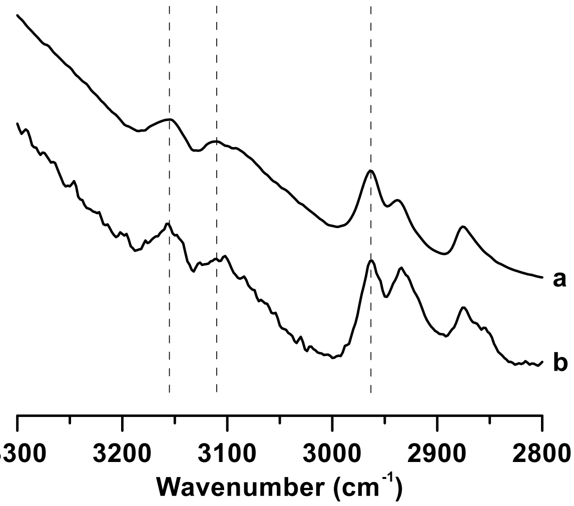

Figure S2. IR spectra of the [C_4MIM][PF_6]/DNA complex obtained at (a) ambient pressure and (b) cycled back to ambient.

© 2019 by the authors. Submitted for possible open access publication under the terms and conditions of the Creative Commons Attribution (CC BY) license (http://creativecommons.org/licenses/by/4.0/).
